# SDF‐1‐edited human amniotic mesenchymal stem cells stimulate angiogenesis in treating hindlimb ischaemia

**DOI:** 10.1111/jcmm.17401

**Published:** 2022-05-26

**Authors:** Hong Zhe Zhang, Seongho Han, Sung‐Whan Kim

**Affiliations:** ^1^ Department of Cardiology The Seventh Affiliated Hospital of Southern Medical University Foshan China; ^2^ Department of Family Medicine College of Medicine, Dong‐A University Busan Korea; ^3^ Institute for Bio‐Medical Convergence, Department Medicine College of Medicine, Catholic Kwandong University Gangneung Korea

**Keywords:** angiogenesis, cell therapy, ischaemia, SDF‐1, stem cells

## Abstract

Although stem cells have extensively been studied as a novel vehicle for tissue repair, their sustained efficacy remains controversial. In this study, we aimed to investigate the angiogenic potency over time of stromal cell‐derived factor‐1 (*SDF‐1*) gene‐edited amniotic mesenchymal stem cells (AMM/S) in a hindlimb ischaemia model. An *SDF‐1* transgene was inserted into the AMM cell genome via transcription activator‐like effector nuclease (TALEN) mediated knock‐in, and cell migration, Matrigel tube formation, and in vivo Matrigel plug assays were performed. AMM/S were also transplanted into hindlimb ischaemia model mice. Blood perfusion, therapeutic potential, histology, capillary density and in vivo angiogenic assays were performed. AMM/S exhibited high expression of the SDF‐1 gene, and robustly promoted migration, proliferation and microvascular formation. AMM/S transplantation significantly increased blood perfusion and limb loss prevention compared with AMM. AMM/S also significantly inhibited increased capillary density and expression of angiogenic factors in the ischaemic hindlimb. Our study demonstrated that AMM/S provides a significant therapeutic effect in ischaemic hindlimbs by enhancing angiogenesis.

## 1 INTRODUCTION

Peripheral artery disease (PAD) is a vascular disease caused by abnormal narrowing of peripheral arteries, restricting blood flow to peripheral tissues, causing pain, ulceration and gangrene. The PAD patient population is estimated to include more than 200 million worldwide. These patients' symptoms usually progress to critical limb ischaemia (CLI) eventually requiring limb amputation.^1^, ^2^ Treatment options for CLI patients are limited primarily to surgical and endovascular procedures. Novel, more effective treatment strategies, including revascularization or therapeutic angiogenesis, are being actively pursued.

Recently, stem and/or progenitor cells have been shown to contribute to therapeutic angiogenesis through paracrine effects (transdifferentiation).^3^, ^4^ Several studies have demonstrated that mesenchymal stem cells (MSCs) can robustly stimulate angiogenesis by secreting angiogenic factors, facilitating favourable therapeutic outcomes.^5^, ^6^ However, the remaining uncertainty in therapeutic outcomes of procedures using stem cells is a major obstacle to their use for damaged tissue regeneration. One interesting angiogenic factor, stromal cell‐derived factor‐1 (SDF‐1), also known as C‐X‐C motif chemokine 12, has been reported to exert potent angiogenic effects. SDF‐1 signals to fibroblasts, osteoblasts and endothelial cells, and plays a key role in cell trafficking and homing of haematopoietic progenitor cells.^7^ SDF‐1 overexpressing rat MSCs promote angiogenesis in a rat model of myocardial infarction.^8^ Thus, SDF‐1 introduction by tissue engineering or transplantation of SDF‐1‐overexpressing cells has been successfully applied to regenerate damaged tissues and organs.^9^

Recently, genome‐editing technologies have been applied to generate functionally improved stem cells.^10^ Such technologies can target specific genomic sites,^11^ and artificial restriction enzymes allow genome modification via homology‐directed repair for knock‐in construction.^12^

In this study, we generated long‐term *SDF‐1* secreting human amniotic mesenchymal stem cells (AMM/S) via TALEN‐mediated editing and investigated to determine whether gene‐edited AMM/S are novel angio‐vasculogenic cell population in experimental ischaemia.

## 2 MATERIALS AND METHODS

### 2.1 Cell culture

Human AMMs were obtained from Thermo Fisher Scientific, Inc. and cultured in low‐glucose Dulbecco's modified Eagle medium (DMEM), supplemented with 10% foetal bovine serum (FBS), 100 U/ml penicillin and 100 μg/ml streptomycin (Gibco). The characteristics of AMMs were described in Figure S1.

### 2.2 Donor construction

SDF‐1 was synthesized and inserted into the AAVS1 genome site targeting donor vector (System Biosciences).

### 2.3 Transfection and selection

Transfection and selection were conducted as described in a previous study.^13^, ^14^ AMMs (1 × 10^5^) were suspended with 0.6 μg of AAVS1 left, right TALE‐Nuclease vector (System Biosciences) and AAVS1 HR Donor (System Biosciences) in 10 μl electroporation buffer, and electroporated using a Neon Transfection System (Thermo Fisher Scientific). Four days after, SDF‐1 knock‐in cells were selected by incubating them in 5 μg/ml puromycin for 7 days.

### 2.4 Fluorescence‐activated cell sorting

After puromycin selection, the cells were washed once with phosphate‐buffered saline (PBS) following the 0.05% trypsin/EDTA treatment for cell detachment. The cells were resuspended in PBS for sorting. AMM/S were sorted on FACSAria™ III Cell Sorter (BD Biosciences).

### 2.5 Genomic DNA extraction and junction polymerase chain reaction

Genomic DNA from cells was extracted using a G‐spin™ Total DNA Extraction Mini Kit (Intron Biotechnology), and 120 ng of genomic DNA was amplified by touch‐down polymerase chain reaction (PCR) (36 cycles) as described in a previous study.^14^ PCR primer was used as following; Forward (J212): aactctgccctctaacgctg, (J195): cgaggccagaggccacttgtgta.

### 2.6 Quantitative reverse transcriptase–PCR (qRT‐PCR) and reverse transcriptase–PCR (RT‐PCR) analyses

qRT‐PCR or RT‐PCR analysis was conducted as described in a previous study.^15^, ^16^, ^17^ Briefly, the total RNA was isolated from cells using RNA‐stat (Iso‐Tex Diagnostics), and RNA was reverse‐transcribed using Taqman Reverse Transcription Reagents (Applied Biosystems). The synthesized cDNA was subjected to qRT‐PCR using human‐specific primers and probes. RNA levels were quantitatively assessed using the ABI PRISM 7000 Sequence Detection System (Applied Biosystems).^14^ The relative mRNA expression normalized to GAPDH expression was calculated as described previously.^18^

### 2.7 qPCR primer

The primers used in qRT‐PCR were for human SDF‐1 (Hs00171022_m1) and GAPDH (Hs99999905_m1) and for mouse fibroblast growth factor‐2 (FGF‐2) (Mm01285715_m1), hepatocyte growth factor (HGF) (Mm01135184_m1), insulin growth factor‐1 (IGF‐1) (Mm00439561_m1), vascular endothelial growth factor A (VEGF‐A) (Mm01281448_g1) and GAPDH (Mm99999915_g1). All of the primer/probe sets were purchased from Applied Biosystems.

### 2.8 Conditioned media preparation

Conditioned media (CM) was prepared as described in previous literature.^19^ Briefly, AMMs, AMM/sh‐S or AMM/S (each 1 × 10^6^) were seeded into T‐75 cell culture flasks and grown in normal medium or low‐glucose DMEM (Gibco) containing 10% FBS, 100 U/ml penicillin and 100 mg/ml streptomycin (Gibco) for 48 h until the cells reached approximately 90% confluence. Each of CM was then centrifuged, and the supernatants were collected and used for this study.

### 2.9 Scratch migration assay

Scratch wound assays were performed by a method established in previous literature^19^ with modifications. Human dermal fibroblast (1 × 10^5^) were seeded in 24‐well culture plates coated with type I collagen (0.2 mg/ml) and incubated at 37°C in 5% CO_2_ to produce confluent monolayers. Monolayers were scratched using a sterile pipette tip and incubated with each CM groups (AMMs, AMM/sh‐S or AMM/S), which had been cultured for 5 days. To measure cell mobility, images were obtained at 5 random fields after scratching. Wound areas were examined, using the NIH Image program (http://rsb.info.nih.gov/nih‐image/).^19^

### 2.10 Matrigel tube formation assay

Each type of AMMs, AMM/sh‐S or AMM/S was seeded with endothelial growth medium (EGM)‐2 (Lonza Inc.) at a concentration of 1.5 × 10^4^ cells/well in basement membrane matrix gel (Matrigel, BD)‐coated 12‐well culture plate. After 12 h of incubation, culture plates were randomly photographed using microscopy. Tube length and the number of branching point from each sample were measured.

### 2.11 In vivo Matrigel plug assay

AMMs or AMM/S with 500 μl of Matrigel were subcutaneously transplanted in Male athymic nude mice (Joongang Laboratory Animal Inc.), aged between 7 and 9 weeks. After 2 weeks, Matrigel plugs were harvested, and the haemoglobin content was measured as previously reported.^20^

### 2.12 Transplantation of cells in the ischaemic hind limb mouse model

All procedures were performed in accordance with the Guide for the Care and Use of Laboratory Animals published by the US National Institutes of Health (NIH Publication No. 85–23, revised 1996). Experimental protocols of animal were approved by the Catholic Kwandong University Institutional Animal Care and Use Committee. Female athymic nude mice (Joongang Laboratory Animal Inc., Seoul, South Korea) aged between 6 and 9 weeks and weighing between 19 and 22 g were used. To induce hind limb ischaemia, the mice were anaesthetised with 2% isoflurane, and their right femoral artery was surgically ligated. 1,1’dioctadecyl‐3,3,3′,3′‐tetramethylindocarbocyanine (Dil)‐labelled 1 × 10^6^ AMM, AMM/S or PBS were intramuscularly injected into the hind limb area after surgery (*n* = 7 for each group).^21^ Immediately before euthanasia, an overdose of pentobarbital (200 mg/kg) was injected into each mouse. We used a laser Doppler perfusion image (LDPI) analyser (Moor Instrument) to measure the serial blood flow in the hind limb after cell injection.

### 2.13 Histological analysis

Mice hind limbs were harvested and fixed for 4 h in 4% paraformaldehyde and incubated overnight in 15% sucrose solution. The tissues were embedded in OCT compound (Sakura Finetek, Torrance, CA, USA), snap‐frozen in liquid nitrogen and sectioned (thickness, 10–20 μm).^22^ Nuclei were counterstained with 4′,6‐diamidino‐2‐phenylindole (1:5000; Sigma Aldrich). For capillary density measurement, five frozen sections of tissue from each group were stained with primary biotinylated ILB4 (1:250; Vector Laboratory Inc.) and secondary strepta‐avidin Alexafluor 488 (1:400; Invitrogen).^21^ Five fields from four tissue sections were randomly selected, and the number of capillaries was counted in each field.^21^ Sections were also labelled with primary antibody to SDF‐1 (Santa Cruz Biotechnology, Inc.) and detected by Cy3 conjugated antibody (Jackson ImmunoResearch Laboratories). Photographs were taken using confocal microscopy (Zeiss LSM 510; Carl Zeiss Inc.).

### 2.14 Statistical analysis

All data were presented as mean ± standard deviation. Statistical analyses were performed with Student's *t* test for comparisons between two groups, and analysis of variance followed by Bonferroni's correction was performed for more than two groups using SPSS version 11.0 (IBM Corp.).^16^, ^17^ A *p* value <0.05 was considered statistically significant. Graphs were drawn using MedCalc software® (Mariakerke, Belgium).

## 3 RESULTS

### 3.1 Targeted *SDF‐1* knock‐in into the AMMs cell genome

**Figure 1:**
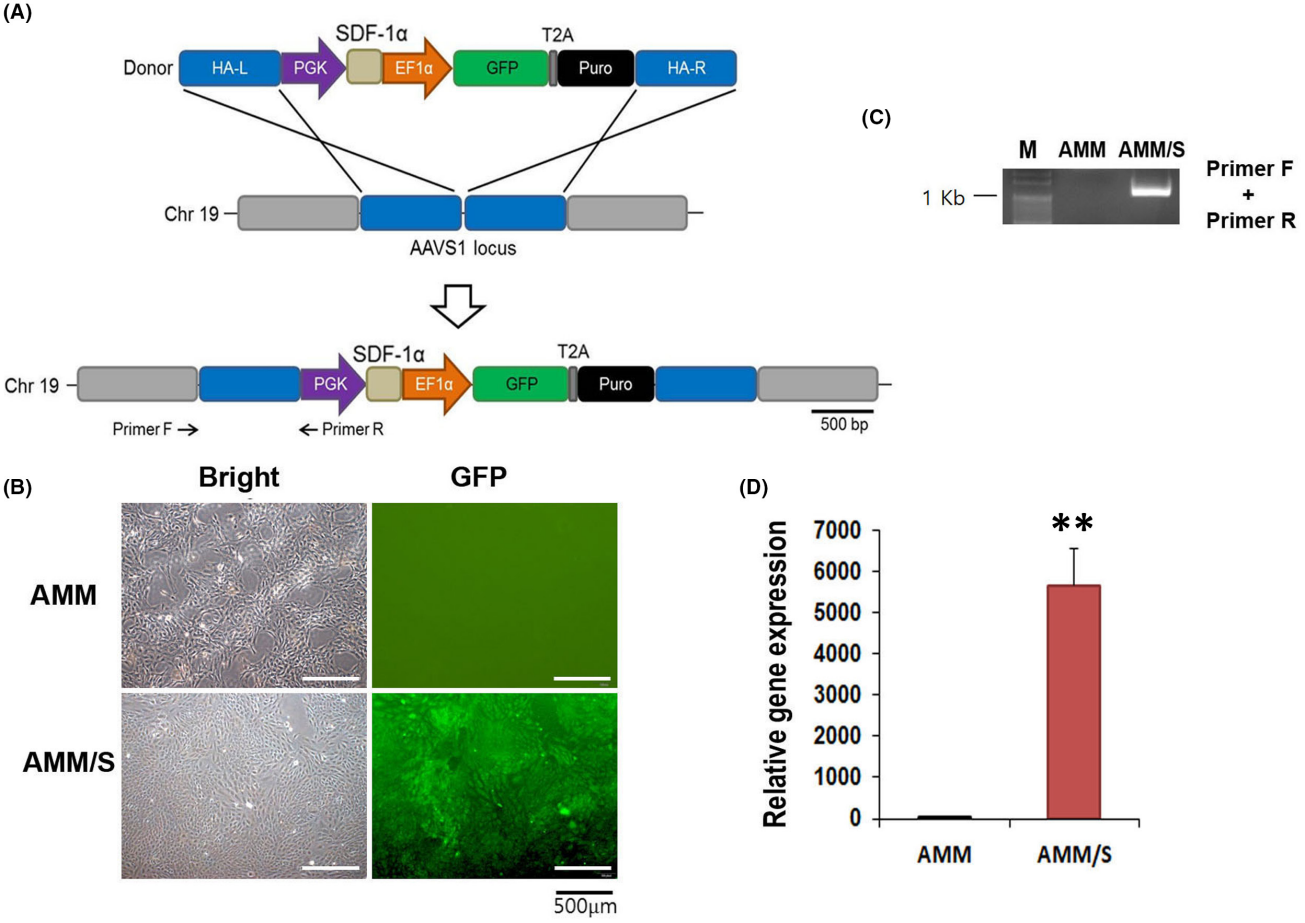
Generation of the AMM/S stable cell line. (A) Schematic of the donor vector carrying SDF‐1 and donor plasmid DNA targeting the AAVS1 locus in the host genome. Primers F and R indicate primer locations for junction detection. Abbreviations: HA‐L, left homology arm; HA‐R, right homology arm; PGK, phosphoglycerate kinase promoter; EF1α, elongation factor‐1 alpha promoter; Puro, puromycin. (B) GFP expression in AMM/S. Transfected cells were selected by incubating with puromycin and then purified by fluorescence‐activated cell sorting. (C) Confirmation of correct knock‐in of donor plasmid into the AAVS1 locus by junction PCR. (D) SDF‐1 expression levels in the AMM/S cell line assessed by RT‐qPCR. **p < 0.01, *n* = 4 in each group

To generate a stable stem cell line expressing *SDF‐1* using the TALEN integration method, we chose a safe locus on chromosome 19 (AAVS1) as the target site. The donor plasmid was designed to carry the PGK promoter, *SDF‐1* and EF1α promoters, and GFP‐T2A‐puromycin (Figure 1A). The donor plasmid was transfected into human AMMs using TALEN. Only 4%–5% of cells were GFP‐positive after transfection. Purity of SDF‐1 knock‐in cells was enriched to approximately 99.1% of GFP‐positive cells by puromycin treatment followed by fluorescence‐activated cell sorting (Figure 1B). To confirm integration of the donor plasmid into the AMM genome, we conducted genomic DNA PCR. Correct insertion of the donor plasmid was confirmed. (Figure 1C). Next, we confirmed *SDF‐1* expression in transfected AMMs by RT‐qPCR. We found that transfected AMMs expressed high levels of SDF‐1 compared to control AMMs, suggesting successful establishment of an *SDF‐1* expressing AMM/S cell line (Figure 1D).

### 3.2 Culture Media (CM) from AMM/S promotes endothelial cell proliferation and migration

**Figure 2:**
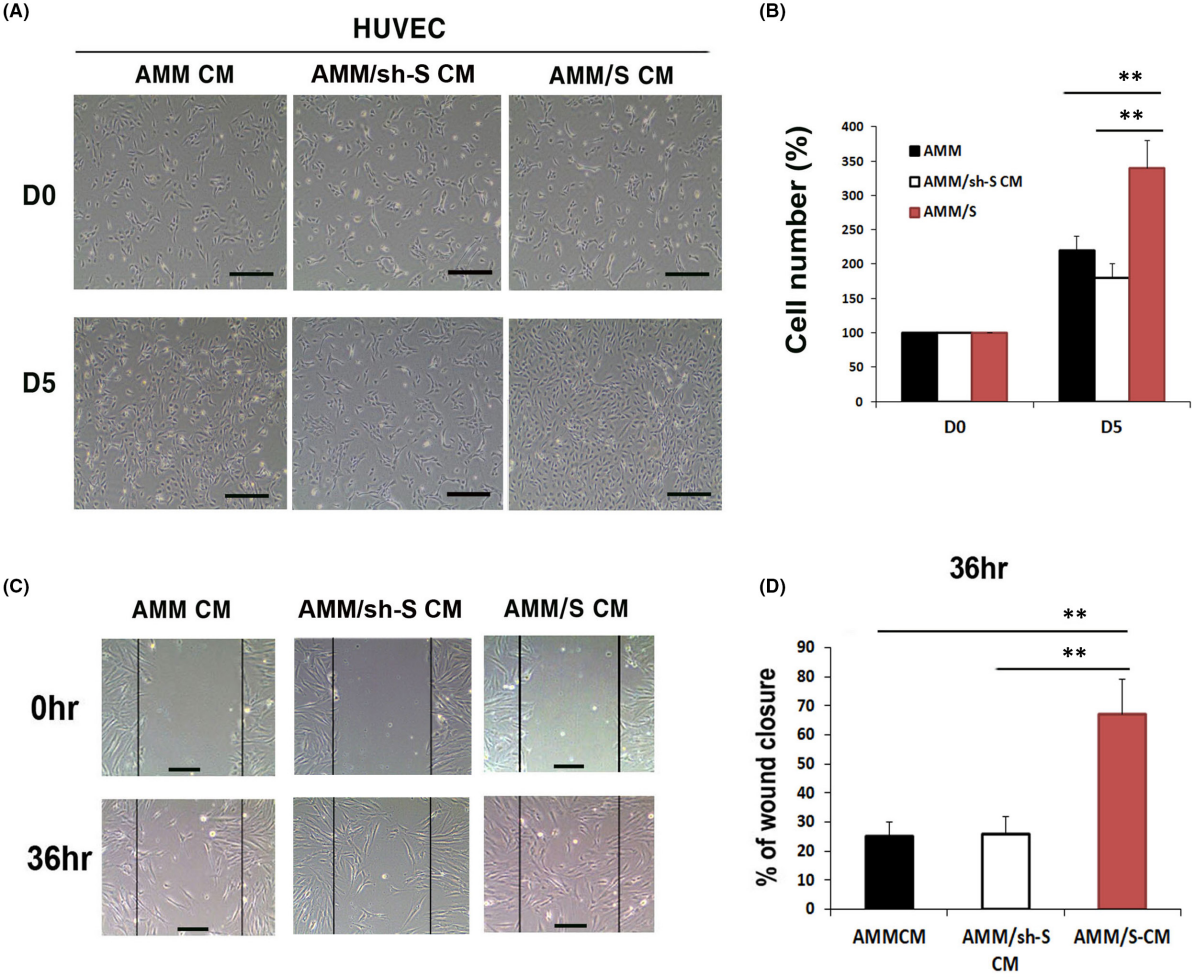
Cell proliferation, migration assays. (A) Representative photograph of HUVEC proliferation after 5 days incubation with CM. (B) Comparison of HUVEC cell proliferation rates after 5 days showed that AMM/S CM significantly improved HUVEC proliferation compared to control AMM or AMM/sh‐S CM. **p < 0.01, *n* = 4 in each group. (C) Representative photograph of HUVEC migration after incubation with CM. (D) An in vitro wound scrach healing assay indicating that AMM/S CM strongly enhanced cells migration compared with control AMM or AMM/sh‐S CM. **p < 0.01, *n* = 4 in each group

To examine whether protein factors secreted from AMM/S promote endothelial cell proliferation and migration, we measured rates of cell proliferation, and performed a scratch migration assay. In addition, to further confirm the function of SDF‐1 in AMMs, we also generated SDF‐1 silencing AMMs using shRNA lentiviral construct (AMM/sh‐S) and AMD3100 (CXCR4 inhibitor) treated AMM/sh‐S used as an another control group. The cell proliferation assay showed that AMM/S CM (333 ± 30%, *p* = 0.001, 0.002) significantly increased rates of HUVEC proliferation compared with control AMMs CM (206 ± 21%) or AMM/sh‐S CM (188 ± 19%) (Figure 2A and B). Scratch closure assay also showed that AMM/S CM (66.3 ± 11%, *p* = 0.001, 0.002) significantly increased rates of wound closure compared with those under exposure to control AMMs (24.3 ± 4%) or AMM/sh‐S CM (24.6 ± 5%) (Figure 2C and D).

### 3.3 AMM/S display angiogenic and vasculogenic properties

**Figure 3:**
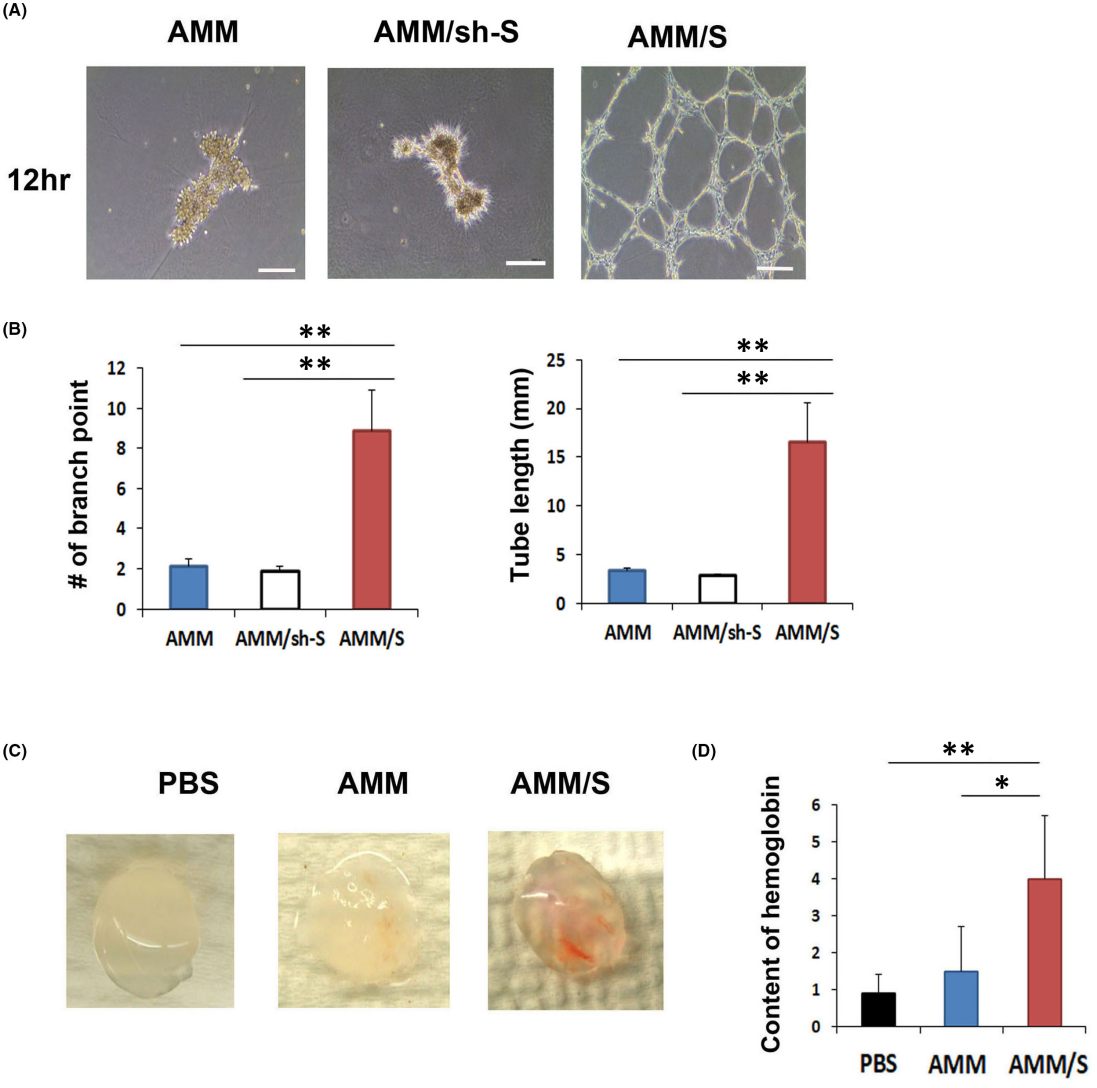
Matrigel tube formation and Matrigel plug assay. (A) Representative photographs of Matrigel tube formation induced by AMM or AMM/S. Bars: 200 μm. (B) Quantification of Matrigel tube formation. Numbers of branching points, and tube lengths were significantly higher with AMM/S than AMM or AMM/sh‐S. **p < 0.01, *n* = 5 per group. (C) Representative photograph of Matrigel plugs injected with AMM/S, AMM or PBS for 1 week. (D) Quantification of haemoglobin content. **p < 0.01; *n* = 5 per group

To investigate vasculogenic potential of AMM/S in vitro, we performed a Matrigel tube formation assay. AMM/S displayed significantly higher tube lengths (8.9 ± 3 mm, *p* = 0.007, 0.006) and branching points (16.6 ± 4, *p* = 0.002, 0.002) than in AMMs (1.8 ± 0.3, 3.2 ± 0.2), or AMM/sh‐S (1.8 ± 0.2, 2.7 ± 0.2) (Figure 3A and B).

To measure the vasculogenic potential of AMM/S in vivo, we performed a Matrigel plug assay. After 2 weeks, Matrigel plugs were harvested, and their haemoglobin content was examined. Interestingly, Matrigel plugs containing AMM/S (4.1 ± 1.5, *p* = 0.02) contained significantly higher levels of red blood cells compared with plugs containing AMMs (1.5 ± 0.6), indicating high potential for inducing formation of vasculature (Figure 3C and D).

### 3.4 AMM/S transplantation exerts strong therapeutic effects in response to hindlimb ischaemia

**Figure 4:**
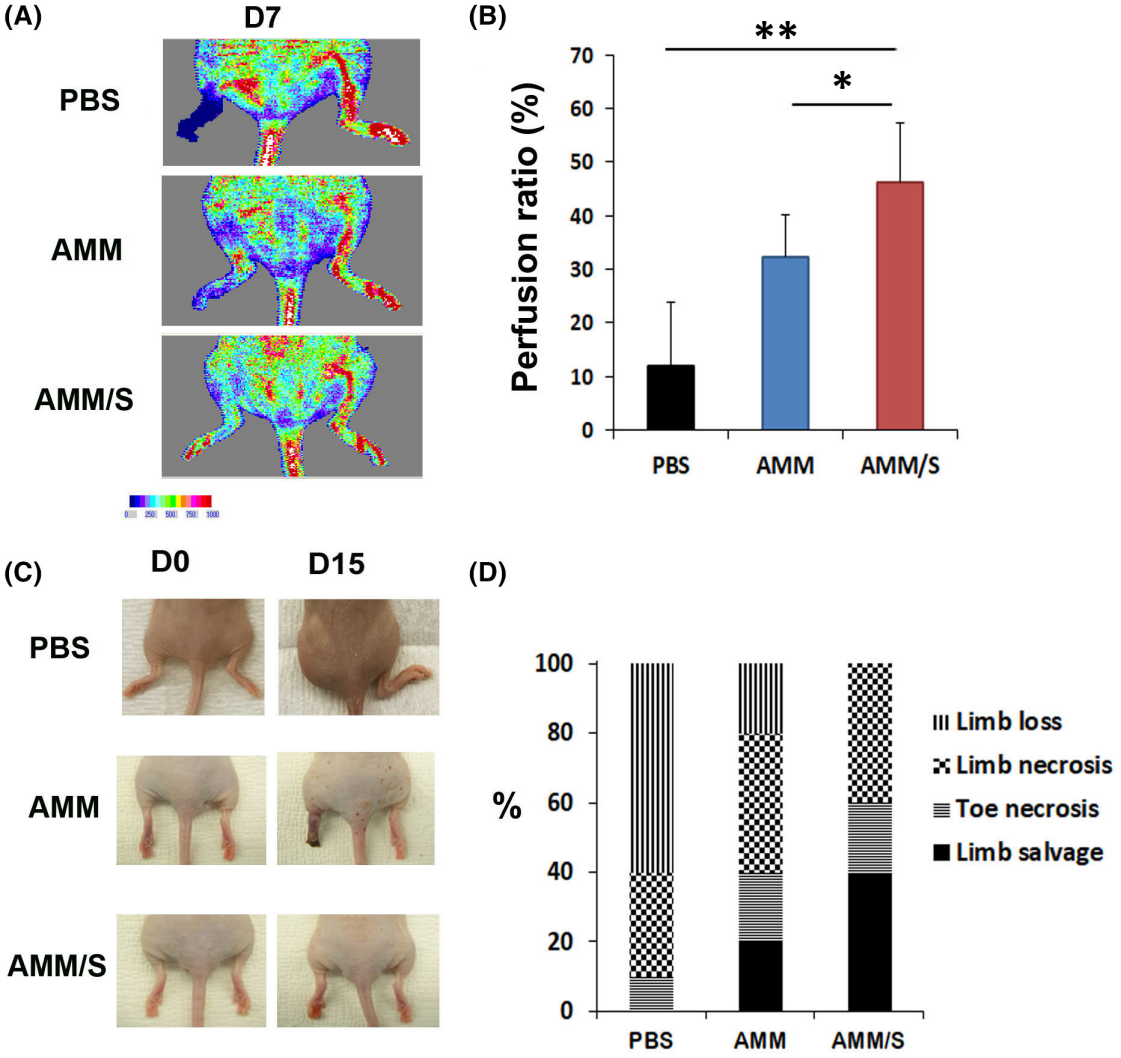
Recovery of blood flow, and therapeutic effects promoted by cell injection in a hind limb ischaemia model. (A) Representative laser doppler images of blood perfusion recovery in ischaemic hind limbs after cell transplantation. (B) Quantitative analysis of blood perfusion ratios. AMM/S injection improved blood flow compared with AMM treated mice 1 week after cell injection. **p < 0.01, *p < 0.05; *n* = 7 per group. (C) Representative images of salvaged limbs after AMM/S injection. (D) Quantitative analysis of limb salvage ratios

We induced hind limb ischaemia in nude mice to examine the therapeutic effects of AMM/S. AMM/S, AMM or PBS were injected intramuscularly into the hind limb. Laser Doppler perfusion image analysis revealed that AMM/S (47 ± 7.1, *p* = 0.03, 0.0006) injection remarkably increased blood perfusion on Day 5 compared with limbs injected with AMM (32 ± 8.6) or PBS (12 ± 3.0) (Figure 4A and B). In addition, the AMM/S‐injected group demonstrated a higher limb function salvage ratio than the AMM and PBS control groups (Figure 4C and D).

### 3.5 AMM/S enhance capillary density in ischaemic hind limb muscle

**Figure 5:**
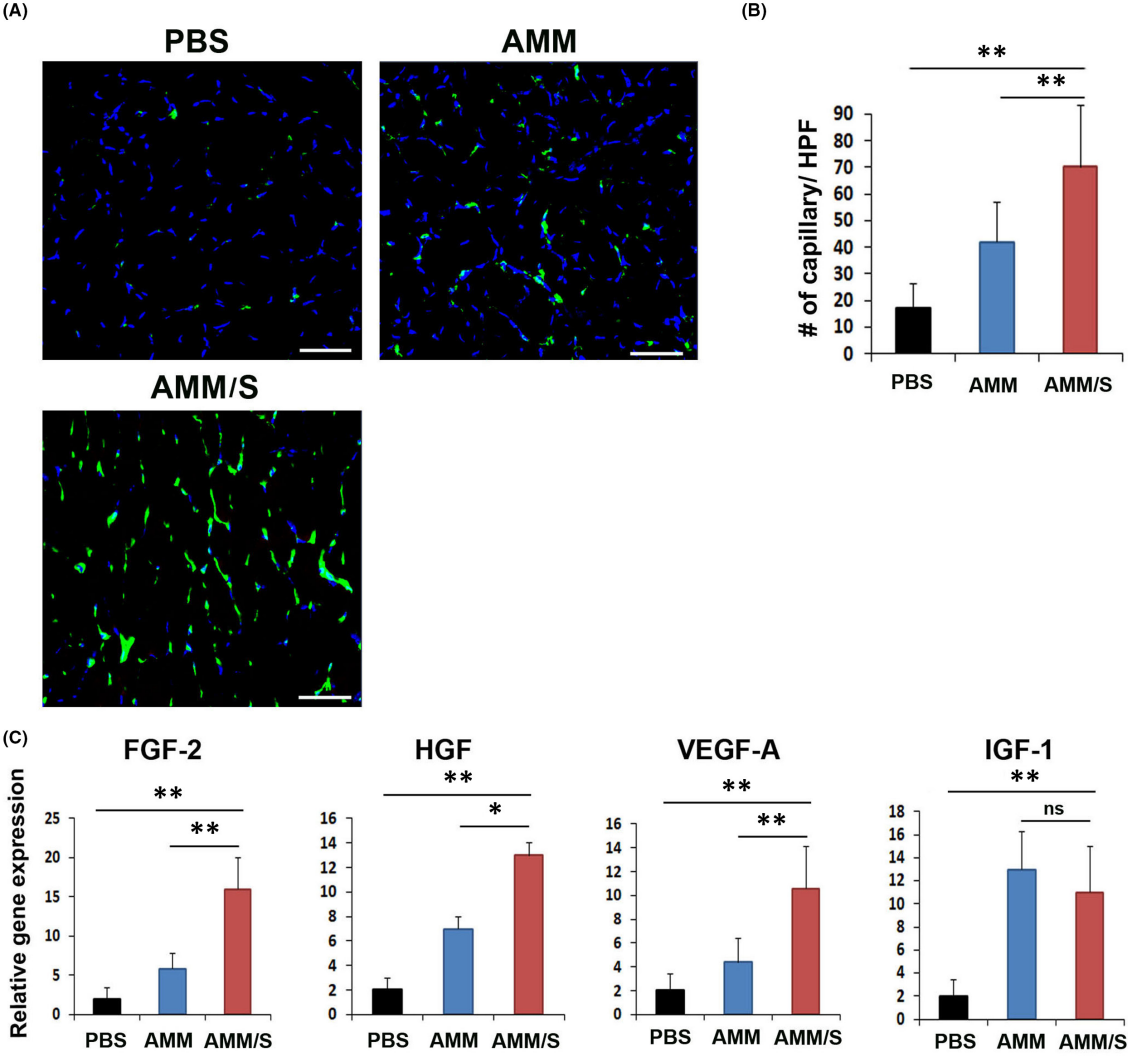
Therapeutic mechanism of hind limb ischaemia after cell injection. (A) Representative images of capillary density in ischaemic hind limbs 2 weeks after cell injection. (B) Quantification of capillary density in ischaemic hind limb tissues after cell injection. **p < 0.01; *n* = 7 per group. (C) Analysis of levels of angiogenic gene expression in ischaemic hind limb tissues 3 days after cell transplantation. Various angiogenic gene expression levels were measured by RT‐qPCR. **p < 0.01; *p < 0.05; *n* = 7 per group

To elucidate the mechanism(s) underlying the therapeutic effects of AMM/S treatment, we harvested hind limb muscle and immunostained tissue sections using SDF‐1 and isolectin B4 (ILB4) as a marker for endothelial cells. The number of ILB4 positive capillaries was significantly higher on Day 7 in the AMM/S‐injected hind limb (67 ± 1.5, *p* = 0.04, 0.003) than in PBS (17.3 ± 5.5) or AMM (44.3 ± 9.7) treated control hind limbs (Figure 5A and B). In addition, immunostaining and Western blot analysis revealed that the expression of SDF‐1(2.8 ± 0.5, *p* = 0.001, 0.001) was higher in AMM/S‐injected hind limb than PBS (1.0 ± 0.3) or AMM (0.9 ± 0.2) treated control hind limbs (Figure S2A,B).

To further elucidate the mechanisms underlying the enhanced recovery of blood perfusion, we examined the expression of angiogenic factors in hind limb tissues via RT‐qPCR analysis. Interestingly, AMM/S injection significantly upregulated FGF‐2 (*p* = 0.001, 0.0003), HGF (*p* = 0.02, *p* = 0.0001), VEGF‐A (*p* = 0.005, *p* = 0.007) compared to AMM or PBS injection five days after cell transplantation, suggesting strong angiogenic stimulation by AMM/S, promoting vascular regeneration (Figure 5C).

### 3.6 Endothelial differentiation potential of AMM/S in vivo

**Figure 6:**
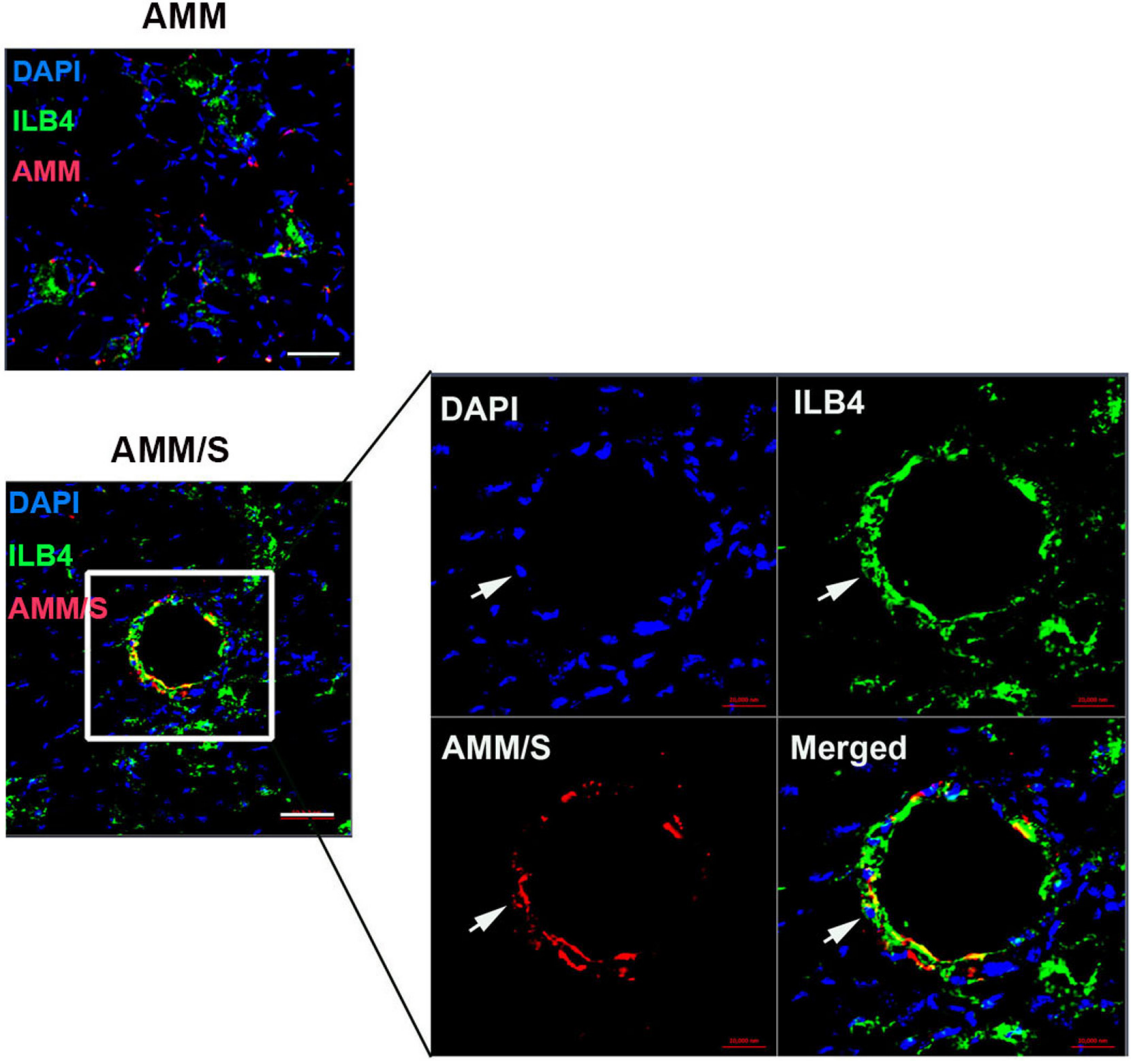
Endothelial differentiation of AMM/S in ischaemic hind limbs. A representative image of vascular‐like structure and transdifferentiated endothelial‐like AMM/S in an ischaemic hind limb 4 weeks after cell transplantation. Arrow indicates AMM/S that colocalize with endothelial marker ILB4

The endothelial differentiation potential of AMM/S was investigated in hind limb ischaemia. Dil‐labelled AMM/S were directly injected into the ischaemic area of the hind limb adductor muscle. Hind limb muscle tissues were harvested 4 weeks after cell injection. Immunohistochemistry was performed, demonstrating that AMM/S colocalized with ILB4 in vascular structure, supporting their endothelial differentiation in vivo (Figure 6). However, we could not find colocalization with ILB4 of AMM in vascular structure.

## 4 DISCUSSION

In this study, we first generated a gene‐edited AMM/S cell line. We also investigated angio‐vascuogenic characteristics of AMM/S and the possible therapeutic mechanism. These results showed that AMM/S injection induced the enhanced recovery of blood flow, neovascularization and prevented limb dysfunction in a mice limb ischaemia model.

Although stem cell‐based cell therapies have emerged as an attractive therapeutic avenue for repair of damaged tissues, the lasting efficacy of its therapeutic effects remains in question.^23^ We initially evaluated allogeneic stem cell source candidates, including adult stem cells, for some important points, including low immunological response, young/unlimited cell proliferation, high transdifferentiation potential and easy accessibility for collection. We identified human amnion as an attractive stem cell resource. We evaluated isolated tissues from the amnion and found the amnion membrane to be an attractive candidate stem cell source. AMM contains abundant cells compared with fluid, cord or chorion, and displays high/unlimited cell proliferation capacity, as previously reported.^24^ Additionally, we tested a strategy to enhance the therapeutic potential of AMMs by genome modification using TALEN gene‐editing methods. Gene‐editing technology can precisely manipulate genes, preventing unintended mutations.

We recently reported angiogenic properties in human AMMs, and ASC overexpressing a single chemokine gene (GCP‐1) displayed high angio‐vasculogenic capacity.^25^ In addition, AMM offers great advantages as an allogeneic stem cell source due to its low immunogenicity, and unlimited cell proliferation capacity.^24^, ^26^ Thus, we hypothesize that one of the representative chemokine genes (SDF‐1) overexpressed in AMM might enhance their angiogenic potential, aiding development of universal stem cell resources. The marginal therapeutic effects of stem cells to date are a major limitation facing cell therapy. In addition, AMM specifically exhibits low SDF‐1 expression compared to other angiogenic factors such as VEGF‐A. In fact, SDF‐1 overexpressing MSC promote angiogenesis and improve heart function.^8^ In line with this report, gene‐edited AMM/S also exhibited strong proliferative and migratory properties. In addition, AMM/S also showed vasculogenic potential in in vitro and in vivo Matrigel assays.

CXC chemokines exert pleiotropic effects on immunity, angiogenesis and cancer metastasis.^27^ In addition, CXC chemokines are a unique family of cytokines that contain the ‘ELR’ motif and potently stimulate angiogenesis via binding and activating CXCR2 on endothelial cells.^27^ SDF‐1, also known as C‐X‐C motif chemokine 12 (CXCL12), induces migration of human umbilical vein endothelial cells in vitro^28^ and enhances formation of tube‐like structures.^29^ Consistent with these studies, SDF‐1 overexpressing AMM/S exhibited strong migration and tube formation properties, suggesting enhanced angiogenic potential.

SDF‐1 exerts a chemoattractive effect on haematopoietic stem cells (HSCs)^30^ and induces bone marrow‐derived c‐kit^+^ progenitor cell differentiation towards an endothelial phenotype.^31^ SDF‐1 pretreatment during endothelial progenitor cell (EPC) expansion stimulates EPC adhesion to endothelial cells, augmenting cell therapy efficacy for ischaemic vascular diseases.^32^ Based on these numerous reports, SDF‐1 affect chemotaxis and differentiation in HSCs and EPCs, enhancing therapeutic angiogenesis in hind limb ischaemia.^8^, ^33^ In addition, MSCs could differentiate into endothelial cells in the presence of SDF‐1.^8^ Consistent with these studies, we found that AMM/S transdifferentiated into endothelial‐like cells with vascular structure, suggesting that autocrine SDF‐1 action might enhance in vivo endothelial cell transdifferentiation in hind limb tissue.

Stem cell therapeutic mechanisms in ischaemic cardiovascular disease have mostly been attributed to paracrine effects.^34^ AMM/S cell injection significantly increased vascular density and resulted in high levels of the proangiogenic factors FGF‐2, HGF, IGF‐1 and VEGF‐A in hind limb tissues. AMM/S injection may stimulate circulating endothelial stem or progenitor cells to home to ischaemic limbs due to high chemokine levels. In addition, in vivo endothelial‐transdifferentiated AMM/S contributed to neovascularization or maintenance of vascular structure in ischaemic limbs.

In conclusion, we demonstrated that SDF‐1 overexpressing AMMs generated by gene‐editing display potent angiogenic and vasculogenic properties. Thus, we speculate that AMM/S may represent a great therapeutic option, as an enhanced stem cell modality of allogeneic therapy in ischaemic vascular diseases.

## AUTHOR CONTRIBUTIONS

**Hong Zhe Zhang:** Data curation (equal); funding acquisition (equal); investigation (equal). **Seongho Han:** Data curation (equal); investigation (equal); project administration (equal); resources (equal); supervision (equal). **Sung‐Whan Kim:** Conceptualization (equal); data curation (equal); funding acquisition (equal); investigation (equal); methodology (equal); project administration (equal); resources (equal); supervision (equal); writing – original draft (equal); writing – review and editing (equal).

## CONFLICT OF INTEREST

The authors declare no conflicts of interest.

## Supporting information


Appendix S1

## Data Availability

Research data are not shared.

## References

[1] Shu J , Santulli G . Update on peripheral artery disease: epidemiology and evidence‐based facts. Atherosclerosis. 2018;275:379‐381. 29843915 10.1016/j.atherosclerosis.2018.05.033 PMC6113064

[2] Frangogiannis NG . Cell therapy for peripheral artery disease. Curr Opin Pharmacol. 2018;39:27‐34. 29452987 10.1016/j.coph.2018.01.005 PMC6019642

[3] Duran JM , Makarewich CA , Sharp TE , et al. Bone‐derived stem cells repair the heart after myocardial infarction through transdifferentiation and paracrine signaling mechanisms. Circ Res. 2013;113:539‐552. 23801066 10.1161/CIRCRESAHA.113.301202 PMC3822430

[4] Kim SW , Houge M , Brown M , Davis ME , Yoon YS . Cultured human bone marrow‐derived CD31(+) cells are effective for cardiac and vascular repair through enhanced angiogenic, adhesion, and anti‐inflammatory effects. J Am Coll Cardiol. 2014;64:1681‐1694. 25323256 10.1016/j.jacc.2014.06.1204 PMC4201782

[5] Lee KD , Kuo TK , Whang‐Peng J , et al. In vitro hepatic differentiation of human mesenchymal stem cells. Hepatology. 2004;40:1275‐1284. 15562440 10.1002/hep.20469

[6] Di Nicola M , Carlo‐Stella C , Magni M , et al. Human bone marrow stromal cells suppress T‐lymphocyte proliferation induced by cellular or nonspecific mitogenic stimuli. Blood. 2002;99:3838‐3843. 11986244 10.1182/blood.v99.10.3838

[7] Aiuti A , Webb IJ , Bleul C , Springer T , Gutierrez‐Ramos JC . The chemokine SDF‐1 is a chemoattractant for human CD34^+^ hematopoietic progenitor cells and provides a new mechanism to explain the mobilization of CD34+ progenitors to peripheral blood. J Exp Med. 1997;185:111‐120. 8996247 10.1084/jem.185.1.111 PMC2196104

[8] Tang J , Wang J , Yang J , et al. Mesenchymal stem cells over‐expressing SDF‐1 promote angiogenesis and improve heart function in experimental myocardial infarction in rats. Eur J Cardiothorac Surg. 2009;36:644‐650. 19524448 10.1016/j.ejcts.2009.04.052

[9] Lau TT , Wang DA . Stromal cell‐derived factor‐1 (SDF‐1): homing factor for engineered regenerative medicine. Expert Opin Biol Ther. 2011;11:189‐197. 21219236 10.1517/14712598.2011.546338

[10] Pollock K , Dahlenburg H , Nelson H , et al. Human mesenchymal stem cells genetically engineered to overexpress brain‐derived neurotrophic factor improve outcomes in huntington's disease mouse models. Mol Ther. 2016;24:965‐977. 26765769 10.1038/mt.2016.12 PMC4881765

[11] Chang HK , Kim PH , Cho HM , et al. Inducible HGF‐secreting Human Umbilical Cord Blood‐derived MSCs Produced via TALEN‐mediated Genome Editing Promoted Angiogenesis. Mol Ther. 2016;24:1644‐1654. 27434585 10.1038/mt.2016.120 PMC5113099

[12] Veres A , Gosis BS , Ding Q , et al. Low incidence of off‐target mutations in individual CRISPR‐Cas9 and TALEN targeted human stem cell clones detected by whole‐genome sequencing. Cell Stem Cell. 2014;15:27‐30. 24996167 10.1016/j.stem.2014.04.020 PMC4082799

[13] Jeong IS , Park Y , Ryu HA , An HS , Han JH , Kim SW . Dual chemotactic factors‐secreting human amniotic mesenchymal stem cells via TALEN‐mediated gene editing enhanced angiogenesis. Int J Cardiol. 2018;260:156‐162. 29506937 10.1016/j.ijcard.2018.02.043

[14] Chae DS , Han S , Lee MK , Kim SW . Genome edited Sirt1‐overexpressing human mesenchymal stem cells exhibit therapeutic effects in treating collagen‐induced arthritis. Mol Cells. 2021;44:245‐253. 33935044 10.14348/molcells.2021.0037 PMC8112166

[15] Kim S‐W , Kim H , Cho H‐J , Lee J‐U , Levit R , Yoon Y‐s . Human peripheral blood‐derived CD31^+^ cells have robust angiogenic and vasculogenic properties and are effective for treating ischemic vascular disease. J Am Coll Cardiol. 2010;56:593‐607. 20688215 10.1016/j.jacc.2010.01.070 PMC2917842

[16] Seo SK , Kim N , Lee JH , et al. beta‐arrestin2 affects cardiac progenitor cell survival through cell mobility and tube formation in severe hypoxia. Korean Circ J. 2018;48:296‐309. 29625512 10.4070/kcj.2017.0119 PMC5889979

[17] Rao Z , Wang S , Bunner WP , Chang Y , Shi R . Exercise induced right ventricular fibrosis is associated with myocardial damage and inflammation. Korean Circ J. 2018;48:1014‐1024. 30334389 10.4070/kcj.2018.0084 PMC6196150

[18] Cho H‐J , Lee N , Lee JY , et al. Role of host tissues for sustained humoral effects after endothelial progenitor cell transplantation into the ischemic heart. J Exp Med. 2007;204:3257‐3269. 18070934 10.1084/jem.20070166 PMC2150988

[19] Kim SW , Zhang HZ , Guo L , Kim JM , Kim MH . Amniotic mesenchymal stem cells enhance wound healing in diabetic NOD/SCID mice through high angiogenic and engraftment capabilities. PloS one. 2012;7:e41105. 22815931 10.1371/journal.pone.0041105 PMC3398889

[20] Kim MS , Kwon HJ , Lee YM , et al. Histone deacetylases induce angiogenesis by negative regulation of tumor suppressor genes. Nat Med. 2001;7:437‐443. 11283670 10.1038/86507

[21] Kim SW , Zhang HZ , Kim CE , An HS , Kim JM , Kim MH . Amniotic mesenchymal stem cells have robust angiogenic properties and are effective in treating hindlimb ischaemia. Cardiovasc Res. 2012;93:525‐534. 22155484 10.1093/cvr/cvr328

[22] Yoon YS , Wecker A , Heyd L , et al. Clonally expanded novel multipotent stem cells from human bone marrow regenerate myocardium after myocardial infarction. J Clin Invest. 2005;115:326‐338. 15690083 10.1172/JCI22326 PMC546424

[23] Shake JG , Gruber PJ , Baumgartner WA , et al. Mesenchymal stem cell implantation in a swine myocardial infarct model: engraftment and functional effects. Ann Thorac Surg. 2002;73:1919‐1925. 12078791 10.1016/s0003-4975(02)03517-8

[24] Tsuji H , Miyoshi S , Ikegami Y , et al. Xenografted human amniotic membrane‐derived mesenchymal stem cells are immunologically tolerated and transdifferentiated into cardiomyocytes. Circ Res. 2010;106:1613‐1623. 20508201 10.1161/CIRCRESAHA.109.205260

[25] Kim SW , Lee DW , Yu LH , et al. Mesenchymal stem cells overexpressing GCP‐2 improve heart function through enhanced angiogenic properties in a myocardial infarction model. Cardiovasc Res. 2012;95:495‐506. 22886775 10.1093/cvr/cvs224

[26] Wolbank S , Peterbauer A , Fahrner M , et al. Dose‐dependent immunomodulatory effect of human stem cells from amniotic membrane: a comparison with human mesenchymal stem cells from adipose tissue. Tissue Eng. 2007;13:1173‐1183. 17518752 10.1089/ten.2006.0313

[27] Strieter RM , Burdick MD , Gomperts BN , Belperio JA , Keane MP . CXC chemokines in angiogenesis. Cytokine Growth Factor Rev. 2005;16:593‐609. 16046180 10.1016/j.cytogfr.2005.04.007

[28] Gupta SK , Lysko PG , Pillarisetti K , Ohlstein E , Stadel JM . Chemokine receptors in human endothelial cells. Functional expression of CXCR4 and its transcriptional regulation by inflammatory cytokines. J Biol Chem. 1998;273:4282‐4287. 9461627 10.1074/jbc.273.7.4282

[29] De Falco E , Porcelli D , Torella AR , et al. SDF‐1 involvement in endothelial phenotype and ischemia‐induced recruitment of bone marrow progenitor cells. Blood. 2004;104:3472‐3482. 15284120 10.1182/blood-2003-12-4423

[30] Wright DE , Bowman EP , Wagers AJ , Butcher EC , Weissman IL . Hematopoietic stem cells are uniquely selective in their migratory response to chemokines. J Exp Med. 2002;195:1145‐1154. 11994419 10.1084/jem.20011284 PMC2193709

[31] De Falco E , Avitabile D , Totta P , et al. Altered SDF‐1‐mediated differentiation of bone marrow‐derived endothelial progenitor cells in diabetes mellitus. J Cell Mol Med. 2009;13:3405‐3414. 20196780 10.1111/j.1582-4934.2009.00655.x PMC4516496

[32] Zemani F , Silvestre JS , Fauvel‐Lafeve F , et al. Ex vivo priming of endothelial progenitor cells with SDF‐1 before transplantation could increase their proangiogenic potential. Arterioscler Thromb Vasc Biol. 2008;28:644‐650. 18239152 10.1161/ATVBAHA.107.160044

[33] Kuliszewski MA , Kobulnik J , Lindner JR , Stewart DJ , Leong‐Poi H . Vascular gene transfer of SDF‐1 promotes endothelial progenitor cell engraftment and enhances angiogenesis in ischemic muscle. Mol Ther. 2011;19:895‐902. 21364544 10.1038/mt.2011.18 PMC3098636

[34] Gnecchi M , Zhang Z , Ni A , Dzau VJ . Paracrine mechanisms in adult stem cell signaling and therapy. Circ Res. 2008;103:1204‐1219. 19028920 10.1161/CIRCRESAHA.108.176826 PMC2667788

