# Supplementary material for: SDF‐1‐edited human amniotic mesenchymal stem cells stimulate angiogenesis in treating hindlimb ischaemia
Source: J Cell Mol Med. 2022 May 26;26(13):3726–35. doi: 10.1111/jcmm.17401 (PMC9258703; doi:10.1111/jcmm.17401)

**Supplementary Data**

**Supplementary materials and methods**

**Flow cytometry**

Passage 4 AMM were suspended in phosphate-buffered saline (PBS) containing 1% bovine serum albumin. Cells were incubated for 20 min with FITC- or phycoerythrin-conjugated monoclonal antibodies specific for CD14, CD29, CD44, CD73, CD90, CD45, CD105 (endoglin), and HLA-DR. All antibodies were purchased from BD Pharmingen (San Diego, CA, USA). Proper isotype-identical IgGs were used as controls. Cells were first stained and then analysed with a flow cytometer (Becton Dickinson, San Jose, CA, USA).

**Western blot analysis.**

Western blot assays were conducted using a previously described method [[1](#_ENREF_1), [2](#_ENREF_2)]. Briefly, protein extracts of each tissues (100 mg each) were separated on 8% SDS–PAGE gels and electrotransferred onto PVDF membranes. The specimens were probed with antibodies against the following: SDF-1 (Abcam, Cambridge, MA, USA) andβb-actin (Santa Cruz Biotechnology, Inc., Santa Cruz, CA, USA). The membranes were washed and incubated with horseradish peroxidase-conjugated secondary antibody, and the signal was detected using an LAS-3000 chemidoc system (Fujifilm, Japan).

Silencing of SDF-1 and treatment of AMD3100

Silencing of SDF-1 was conducted by previously described method [[3](#_ENREF_3)] . For the silencing of SDF-1, target sequence CATCAGTGACGGTAAACCAGTC (consortium number TRCN0000195944), or a scrambled (SCR) sequence, was cloned into the pLKO.1-puro vector (Sigma, St. Louis, MO, USA), and lentiviral particles were generated using a packaging mix (Sigma) in human 293T cells. The AMM were infected with the lentiviral particles, and selected by using puromycin (10 μg/ml). For the inhibitor treatment, cells were preincubated with AMD3100 (100ng/ml; Sigma) for 1 hour at 37 °C in a 5% CO2 incubator. After treatment with the indicated inhibitor in each group and dissociation into single cell suspension and used for the study.

**Supplementary Figure Legend**

**Supplementary Figure 1.** Characteristics of AMM. (A) Microscopic view of AMM (passage 4). Bar: 100μm. (B) Growth curves of AMM during cell culture. AMM underwent more than 50 population doublings. (C) Representative FACS surface markers of AMM exhibiting MSC-specific cell surface markers. FACS analysis showed that AMM minimally expressed the haematopoietic cell markers (CD14 and CD45) and MHC class II molecules (HLA-DR) and high levels of the MSC-specific markers CD29, CD44, CD73, CD90, and CD105 Green colour represents the isotype control and red represents the specific monoclonal antibody.

**Supplementary Figure 2. SDF-1 expression in tissue after cell transplantation.** (A) Immunocytochemistry results in hindlimb tissues after cells transplantation. DAPI (Blue fluorescence), SDF-1 (Red fluorescence). Bars: 500μm. (B) Western blot results of the expression of 5 representative hepatocyte-specific proteins. Protein levels of AFP, ALB and HGF correlated with the microarray data.

**Supplementary Figure 1**

**
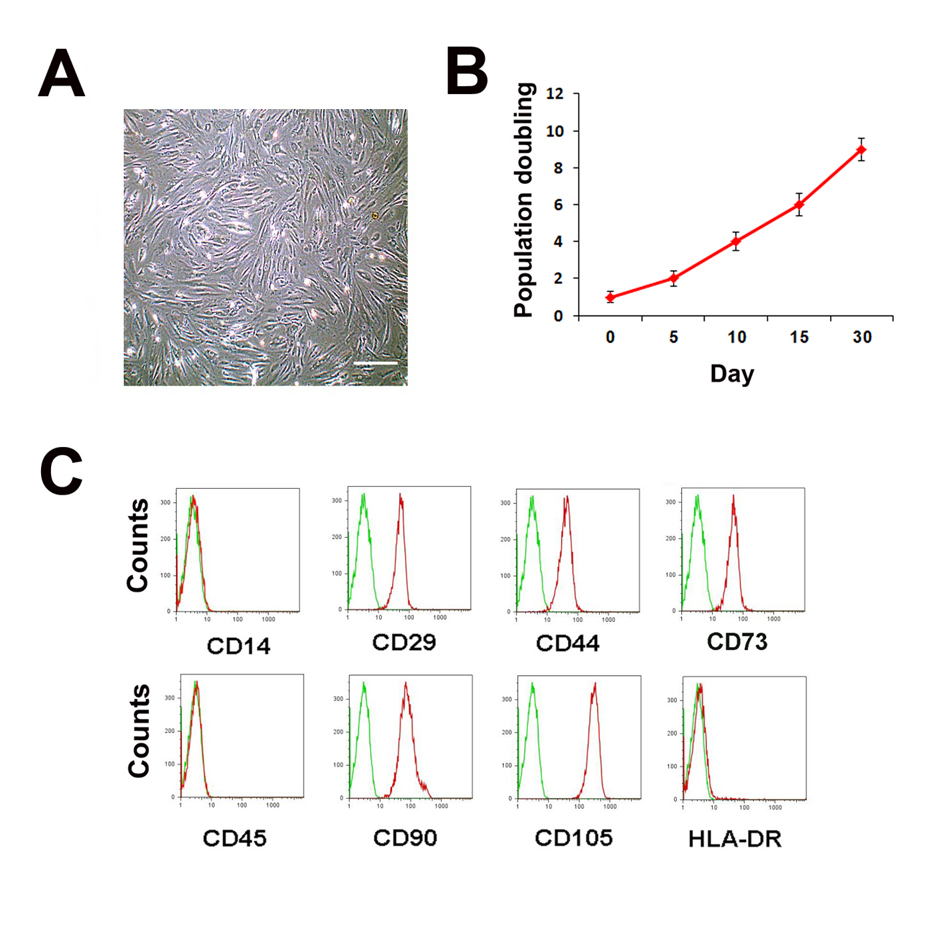
**

**Supplementary Figure 2**


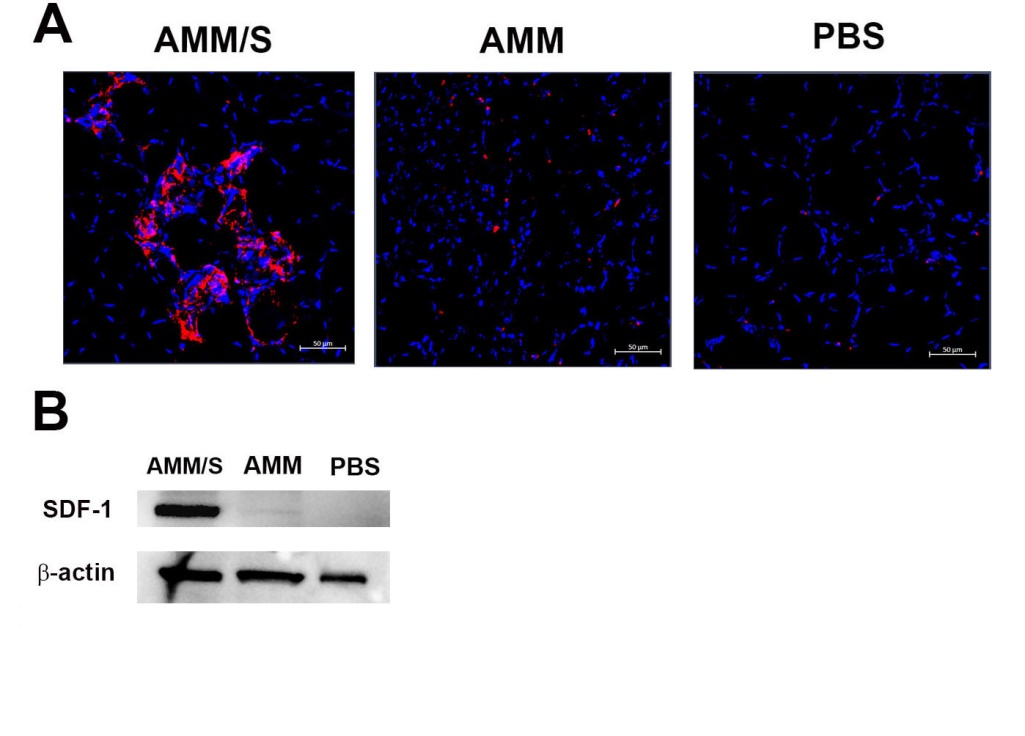


**References**

[1] Youn YJ, Yoo BS, Son JW, Lee JW, Ahn MS, Ahn SG, et al. Remote Ischemic Conditioning by Effluent Collected from a Novel Isolated Hindlimb Model Reduces Infarct Size in an Isolated Heart Model. Korean Circ J. 2017;47:714-26.

[2] Eom YW, Jung HY, Oh JE, Lee JW, Ahn MS, Youn YJ, et al. Isoproterenol Enhances Tumor Necrosis Factor-Related Apoptosis-Inducing Ligand-Induced Apoptosis in Human Embryonic Kidney Cells through Death Receptor 5 up-Regulation. Korean Circ J. 2016;46:93-8.

[3] Kwon MY, Ghanta S, Ng J, Tsoyi K, Lederer JA, Bronson RT, et al. Expression of Stromal Cell-Derived Factor-1 by Mesenchymal Stromal Cells Impacts Neutrophil Function During Sepsis. Critical care medicine. 2020;48:e409-e17.

**Images of gel electrophoresis or blots**

**Figure 1B. Images of gel electrophoresis**


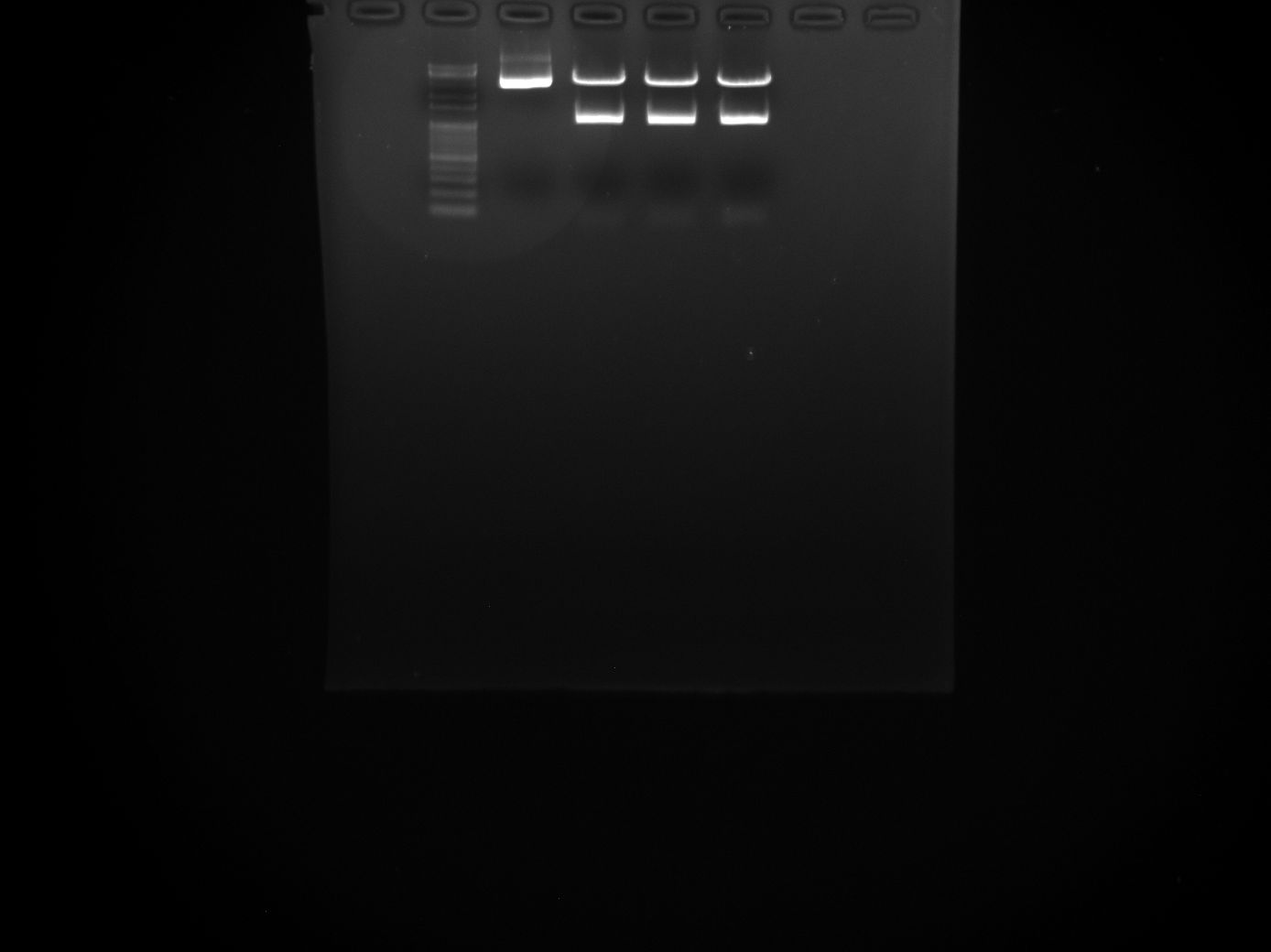


**Figure 1B. Images of western blot**


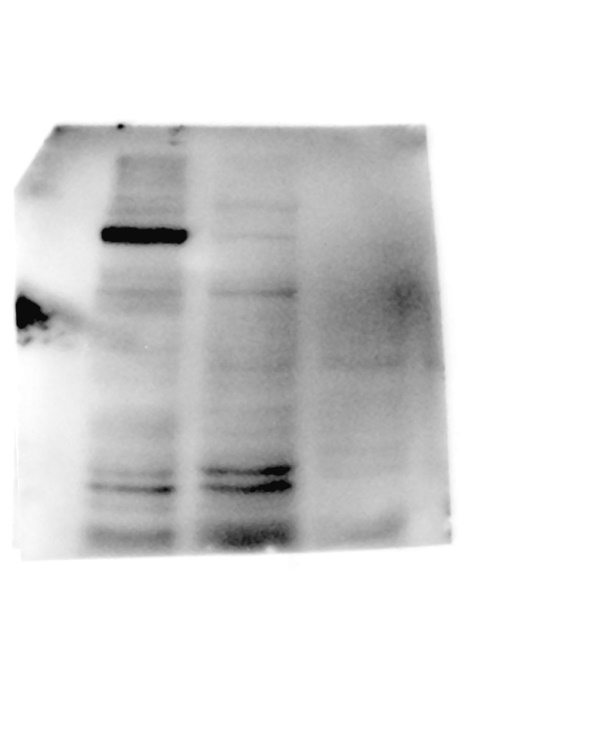

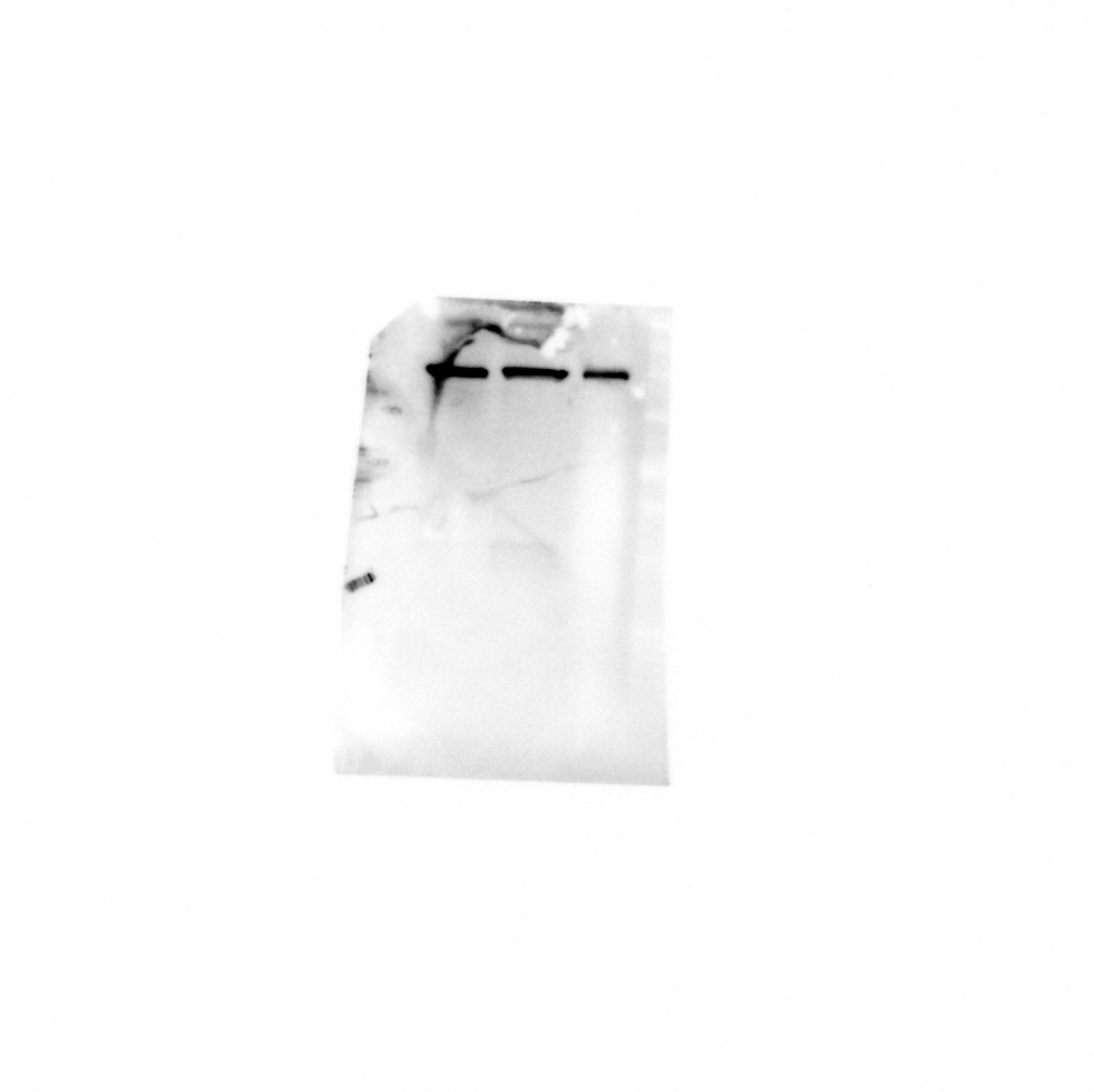

Supplement: Supplementary file 1 — Appendix S1 [file JCMM-26-3726-s001.docx]
